# The ontogeny of exploratory object manipulation behaviour in wild orangutans

**DOI:** 10.1017/ehs.2021.34

**Published:** 2021-07-02

**Authors:** Caroline Schuppli, Anaïs Van Cauwenberghe, Tatang Mitra Setia, Daniel Haun

**Affiliations:** 1 Development and Evolution of Cognition Research Group, Max Planck Institute for Animal Behavior, Bücklestrasse 5a, 78467 Konstanz, Germany; 2 Department of Anthropology, University of Zürich, Winterthurerstrasse 190, 8057 Zürich, Switzerland; 3 Department of Biology, Graduate School and Faculty of Biology, Universitas Nasional, Jl. Sawo Manila, RT.14/RW.3, Ps. Minggu, DKI Jakarta, Indonesia; 4 Max Planck Institute for Evolutionary Anthropology, Deutscher Platz 6, 04103 Leipzig, Germany

**Keywords:** exploration, object manipulation, orangutans, cognitive development

## Abstract

In human infants, exploratory object manipulation is a major vehicle for cognitive stimulation as well as an important way to learn about objects and basic physical concepts in general. The development of human infants’ exploratory object manipulation follows distinct developmental patterns. So far, the degree of evolutionary continuity of this developmental process remains unclear. We investigated the development of exploratory object manipulations in wild orangutans. Our data included 3200 exploration events collected on 13 immatures between the ages of 0.5 and 13 years, at the Suaq Balimbing monitoring station in Indonesia. Our results identify several parallels between the development of exploratory behaviour in humans and orangutans: on top of a highly similar overall age trajectory, we found an increase in variability of the actions used, an increase in the number of body parts involved in each event, and an overall decrease of mouthing of the objects. All in all, our results show that orangutans progress through a developmental sequence of different aspects of exploration behaviour. In combination with previous findings from captivity, our results also provide evidence that exploratory object manipulations reflect cognitive development and might function as a means of cognitive stimulation not just in humans but across the great apes.

## Graphical abstract

**Figure:**
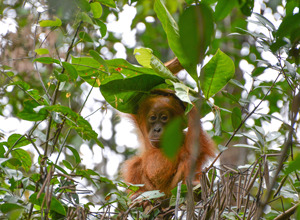

**Social media summary:** Young orangutans go through a distinct developmental sequence of exploratory object manipulation behaviour resembling the human pattern.

## 1. Introduction

Human infants learn about the physical properties of the world around them through exploration, i.e., the deliberate manipulation and visual investigation of objects (Fenson & Schell, 1985; Muentener et al., 2018; Power, 1999; Ruff et al., 1992). By examining objects, human infants gain knowledge about their properties as well as about the general principles of the physical world linked to them (Baldwin et al., 1993; Baumgartner & Oakes, 2011, 2013; Bourgeois et al., 2005; Fenson & Schell, 1985; Power, 1999). Exploratory object manipulation is also an important vehicle of cognitive stimulation for human infants (Piaget, 1962). Through the exploration of objects, developing human infants gain a greater knowledge of affordances and increased causal understanding of their properties which can be transferred to other contexts. Ultimately, this latent learning process widens the pool of knowledge which individuals can draw from when confronted with novel problems (Byrne, 2016; Greenberg & Mettke-Hofmann, 2001; Lockman, 2000; Oakes & Baumgartner, 2012; Piaget, 1962; Power, 1999). Accordingly, in humans, exploratory tendency is highly predictive of current and future cognitive performance, suggesting that exploratory object manipulation is not just a manifestation of infants’ curiosity about their environment but also of their advancing cognitive development (Belsky & Most, 1981; Caruso, 1993; Piaget, 1962).

Since the 1950s, it has generally been acknowledged that, just like humans, many animal species have an exploratory drive that acts independently of immediate stimulation or biological needs (Harlow et al., 1950; Montgomery, 1954; Power, 1999). Just like in humans, exploration in animals seems to serve to learn about objects (Bard, 1995; Bruner, 1972; Chevalier-Skolnikoff, 1989; Greenberg, 2003; Hayashi et al., 2006; Lamon et al., 2018; McGrew, 1977; Tayler & Saayman, 1976). Across species, exploratory object manipulation is readily associated with, and certainly the key feature of, all independent learning (Fenson & Schell, 1985; Heyes, 2012; Muentener et al., 2018; Piaget, 1962; Power, 1999; Ruff et al., 1992). Most forms of social learning (including many forms of observational social learning) also entail a phase in which the learner is trying out and practising the observed skills (Galef, 2015; Heyes, 2012; Piaget, 1952; Reader & Laland, 2002; Schuppli, Meulman, et al., 2016; Whiten, 2015). Therefore, in immatures across species, exploratory object manipulation is most likely part of virtually all learning about the physical world and thus arguably an important mechanism for the translation of immatures’ cognitive potential into actual skills and knowledge. These skills include the basic handling of ecologically relevant objects, such as food items or nesting substrate and more complex skills such as the ones involved in extractive foraging or tool use. The knowledge gained through exploratory object manipulation includes physical properties of the involved objects and the general physical principles of the world around them (e.g., gravity, motion and weight).

Studies across a wide range of different primate and non-primate species also suggest positive inter- and intraspecies correlations between exploratory tendency, problem-solving ability, and innovation probability (Auersperg et al., 2011; Benson-Amram et al., 2016; Griffin & Guez, 2014; Overington et al., 2011; Reader & Laland, 2002; Webster & Lefebvre, 2001). The pattern of increasing exploration propensity across those species correlates with the evolution of increasing brain size and advancing cognitive capacity as well as the behavioural expressions of those, such as tool use and other complex foraging behaviours (Boesch & Boesch, 1993; Byrne & Byrne, 1993; Gibson, 1986; Heldstab et al., 2016; Leca et al., 2011; Meulman et al., 2012, 2013; Schuppli, Graber, et al., 2016; van Schaik et al., 1999).

Because of the apparent significance of exploratory object manipulation for human cognitive development, a considerable amount of research has been carried out on the detailed nature and developmental changes of human infants’ exploration behaviour. Exploratory tendency, in the form of visual, manual and oral manipulation of objects**,** peaks around the age of 2 years and then decreases (Belsky & Most, 1981; Bock, 2005; Power, 1999; Power et al., 1985). In general, human infants explore less with increasing exposure time to and with increasing familiarity with the object (Ruff et al., 1992). Whereas early exploration behaviour is characterized by single actions, with increasing age**,** the behaviour becomes more complex (i.e., a larger numbers of different types of actions are being combined, at first randomly and ultimately in meaningful sequences; Belsky & Most, 1981; Fenson & Schell, 1985; McCall, 1974; Power, 1999). Several studies found that the length, diversity, concentration, and efficiency of human infants’ exploratory actions increases with age (Belsky et al., 1980; McCall, 1974; Muentener et al., 2018; Power, 1999). However, other studies did not find such effects (Palmer, 1989; Power et al., 1985). At the same time, whereas in early years, exploratory mouthing is the predominant form of exploration, the use of the fingers becomes increasingly prominent with increasing age (Belsky & Most, 1981; Fenson & Schell, 1985; Power, 1999; Ruff et al., 1992). Furthermore, the exploratory drive of human infants gets significantly shaped by social inputs during development (Bakeman et al., 1990; Belsky et al., 1980; Main, 1983; Schuetze et al., 1999).

Intriguingly, the developmental sequence through which human infants transition when developing their exploratory behaviour to some degree resembles the phylogenetic scale across species. In primates, an increased and more flexible exploratory tendency seems to be more prevalent than in other taxa (Power, 1999; Vauclair, 1984), even though it remains unclear if cognitive or morphological differences (such as the presence or absence of hands) or a combination of both underly this pattern. However, there seems to be a clear trend within the primate lineage: from strepsirrhine Primates to Old- and New World monkeys to apes, exploratory tendency increases (Vauclair, 1984), manipulations get more varied (Heldstab et al., 2016; Vauclair, 1984), involve more different body parts, and more often involve fingers as opposed to the mouth (Power, 1999; Ruff et al., 1992; Vauclair, 1984). Humans’ closest relatives, the non-human great apes (henceforward called great apes), show the highest levels of exploratory tendency, including the most flexible, varied and complex manipulations (such as object–object or object–substrate combinations, bimanual manipulations and manipulations with a larger number of body parts involved) of all non-human primates (Byrne et al., 2001; Hayashi & Matsuzawa, 2003; Heldstab et al., 2016; Power, 1999; Torigoe, 1985), even though they are less complex than manipulations seen in humans (e.g., human infants show a richer and more differentiated repertoire of manipulations, including more object–object combinations and bimanual manipulations as well as more frequently extracting objects from their backgrounds than great ape infants; Redshaw, 1978; Vauclair & Bard, 1983).

A so far unsolved question is how different aspects of exploratory tendency develop in detail in wild non-human primates. The development of an exploratory tendency and its variation at the individual level may help to further elucidate the importance of developmental inputs for great ape exploration. Similarities in the overall development of exploratory behaviour between humans and great apes would also support the hypothesis that our exploratory drive evolved from a common ancestor. Studies that have looked at the exploratory tendency of captive great apes support a strong effect of the individuals’ developmental histories on the exploratory tendency (Bard & Gardner, 1996; Call & Tomasello, 1996; Hayashi & Matsuzawa, 2003). Recent studies have also shown that in captive orangutans (*Pongo* spp.), the variability and persistence of exploration behaviour are highly correlated with problem-solving success (Damerius, Graber, et al., 2017) and are best predicted by an individual's rearing history and past experience level of human contact (Damerius, Forss, et al., 2017). A handful of studies on the ontogeny of tool use behaviour in non-human primates have looked in detail at how the underlying manipulations develop in individuals. These studies found that the rates of manipulations involved in tool use increase with age and that appropriate sequential combinations of the involved manipulations and objects (i.e., as they are performed during the actual tool use behaviour) occur later than the individual manipulations (De Resende et al., 2008; Inoue-Nakamura & Matsuzawa, 1997; Tan, 2017).

Comparison of wild and captive non-human primates shows that captivity significantly increases overall exploratory tendency (Forss et al., 2015; Kummer & Goodall, 1985). This has been linked to the fact that (mainly because they don't have to find food) captive individuals have increased amounts of time and energy available for exploration compared with their wild conspecifics (Kummer & Goodall, 1985). Furthermore, throughout development captive subjects may be provided with different availabilities of objects as well as different means of interacting with those objects than they would experience under natural conditions (e.g., through increased time they spend on the ground and thus with both hands available for manipulating objects).

Because exploratory behaviour is highly dependent on environmental conditions and has a strong age dependency, to understand natural exploratory behaviour and its function in everyday learning, we have to look at the development of exploration behaviour in wild great apes across age. So far, the number of studies that have looked at the development of exploration behaviour in wild great apes remains limited (but see: Bard, 1995; Koops, Furuichi, & Hashimoto, 2015; Koops, Furuichi, Hashimoto, et al., 2015; Lamon et al., 2018; Schuppli et al., 2017). In a previous study on wild orangutans, we found that exploratory object manipulation is heavily socially mediated: on the proximate level, being in association with conspecifics increases rates of exploration behaviour in adults and juveniles (Schuppli et al., 2017). On the developmental level, the amount of past experienced sociability predicts later exploratory tendency in immature orangutans (Schuppli et al., 2020). Interestingly, adults and juveniles in more sociable populations are more exploratory even when on their own compared with individuals in less sociable populations (Schuppli et al., 2017).

The current study aimed at looking into the development of the natural exploration behaviour of wild orangutans in more detail, using a longitudinal and cross-sectional study design. Specifically, we wanted to find out to what extent the patterns that have been established for the development of an exploratory tendency in humans can also be found in immature orangutans. Orangutans are especially suitable for examining immature animals’ exploration behaviour because of their long developmental period and advanced cognitive skills (Damerius et al., 2019; van Noordwijk et al., 2018). In terms of the overall developmental trajectory of the behaviour, we predicted that (a) immature orangutans show the highest rates of exploratory object manipulations during the early dependency period, just as has been found for humans and wild chimpanzees (*Pan troglodytes*). Based on the human data we also hypothesized that, as in humans, with increasing age, immature orangutans will explore more persistently and more diversely. We therefore predicted that, with increasing age, (b) exploration events would become longer and that (c) exploratory object manipulations would become more diverse in terms of the number of different actions performed and the number of body parts involved, with less overall involvement of the mouth.

## 2. Methods

### 2.1 Data collection

The data for this study were collected from 2007 to 2019 in wild Sumatran orangutans (*Pongo abelii*) at the Suaq Balimbing research site located in South Aceh, Indonesia.

Exploration was defined as prolonged, non-repetitive, usually destructive manipulations of objects, whereby the visual and tactile foci of the individual are on the object. The set minimal duration of an exploration event to qualify as such was 5 seconds. The duration of each event was defined as lasting from the beginning of the manipulation activity until the point in time when the individual had stopped manipulating the object for at least 10 seconds. Following Pisula (2008), we excluded feeding (defined as such by actual ingestion) and object play that did not have any explorative elements (i.e., manipulations where the visual and tactile foci were desynchronized, and were characterized by repetitive movements) from exploration. This approach includes all exploratory object play but not non-exploratory play manipulations which are thought to be separate constructs (Hutt, 1966; Pellegrini & Gustafson, 2005).

Focal animals were 13 immature orangutans (see Table S4), aged from 0.5 to 13.1 years. The focal animals were divided into dependent immatures (immatures that are still suckling, 0–8 years of age) and independent immatures (immatures that have stopped suckling but are not sexually active yet, 8.1–13.5 years of age).

Exploration events were collected by experienced observers during nest-to-nest focal animal follows and on an all-occurrence basis. To look at the overall exploratory tendency, we calculated exploration rates as a function of age. For this, we used data collected by CS, AVC and seven additional, highly trained observers. All observers had passed a 90% inter-observer reliability test on occurrence rates with CS for the specific detailed age classes on which their data was used (young dependent immatures, 0–5 years; old dependent immatures, 5.1 to weaning; juveniles, weaning to adulthood). To be able to assess age-specific developmental states of immature individuals and reliable rates of rare behaviours, we aimed to follow immature individuals for a minimum of 4 days within 6 months. This approach divided our data into so-called ‘data blocks’. Each data block spanned over a period of maximally five months (4–156 days, mean = 54 days) and contained at least 20 (20–131, mean = 87) follow hours. Data blocks on the same individual were separated by 223–1250 days (mean = 775 days). Exploration rates were calculated based on data collected on one individual during each data block available on this individual. The total sample for this analysis contained 23 exploration rates by 12 individuals (five males and seven females) based on a total of 3202 exploration events, whereby each individual immature contributed to one to six (mean = 1.9) age–individual data blocks and exploration rates (see Table 3 for details on the focal individuals).

To look at the nature of the exploration events in more detail, we focused on the age period with the highest exploration rates and thus densest data, namely the dependency period (0–8 years; see Table 3 for details on the focal individuals). From 2013 to 2019, exploration events collected by CS and AVC were timed and described in detail in terms of the object involved, body parts used for the manipulation and the different manipulative actions that were performed. The two observers reached an interobserver reliability of 85% on occurrence rates of the body parts and explorative actions involved in the exploration events, assessed during two simultaneous full-day follows at the end of AVC's data collection training period. Furthermore, there was no statistically significant difference in the duration of the exploration events recorded by the two observers. We had detailed information available for 2430 exploration events by seven dependent immatures (five males and two females). For the analyses with this dataset, we used daily averages. However, to avoid issues of pseudo replication and autocorrelation, we also divided this data into data blocks, following the same criteria as above and added the ID of the block as a random factor in the analyses (see below). We only included blocks which contained a minimum of 50 (50–488, mean = 203) exploration events. Each focal animal contributed to one to three (mean = 1.7) of these data blocks (one individual was excluded owing to there being too few follow hours).

Two exploration events had to be separated by at least 10 seconds during which the focal animal engaged in a different activity to be counted as two events. To assess exploratory diversity, we counted the number of actions performed with the object per exploratory event (i.e., exploratory action diversity; see Table S1 for a list of all explorative manipulations observed in this study and Table S4 for their combinations) and the number of body parts that were used for the manipulation (see Table S2 for a list of all body parts used during the explorative manipulations observed in this study). The use of the mouth during and exploration event was considered as exploratory mouthing.

### 2.2 Statistical analyses

We used the R programming language to analyse and visualize these data (R Development Core Team, 2019). To assess the effects of age on exploration duration, exploration variability (defined as the average number of explorative actions performed by event and number of body parts used for each event), and the share of exploration events that involved mouthing, we computed average daily values. For the exploration duration, we used linear mixed-effects models (implemented in the *lme4* package in R; Bates et al., 2011). For the count data (exploration variability and share of mouthing events), we used generalized mixed models with a Poisson family distribution whereby we used the total number of exploration events as an offset. As a first step, to assess the effect of age, we compared our full models (including age as a predictor variable) with a null model (which only included the random effects), using a likelihood ratio test (LRT) via the *anova* function (Dobson & Barnett, 2018; Fox, 2015). Because, for some of our response variables, visual inspection of the age effect suggested a flattening after an initial increase over age, we additionally tested if including age as a sigmoid factor (using the *sigmoid* package in R; Quast, 2018) would further increase the model fit, again using likelihood ratio tests. Aside from age, we also tested for sex differences by including sex as an additional factor in each model, using likelihood ratio tests. However, including sex did not significantly improve the fit of any of the models in any of the analyses and it was thus not included in the final models. To account for the fact that several individuals occurred multiple times in the dataset, we included the individual as a random effect in the analyses. To avoid autocorrelation and pseudo replication issues caused by data points that were collected close together in time, the ID of the block in which the data of each data point was collected (see above) was included as a random effect in all analyses. We report the results of the model comparisons and the details on the best fitting models. All model fits were examined visually to assess whether they satisfied model assumptions and to check for the presence of influential observations (Harrell, 2015), and in the case of the Poisson models, overdispersion (Mundry, 2014). For the plots, the mean age of each data block was computed (weighted according to the age of the focal individual at each day of data collection).

## 3. Results

### 3.1 Overall developmental trajectory of exploration behaviour

**Figure 1.**
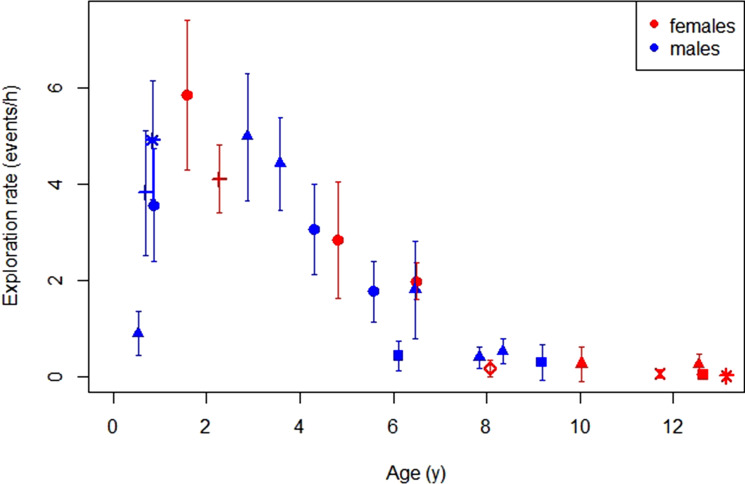
Development of exploratory tendency: average hourly exploration rates over age for immature females and males, based on the age-individual data blocks. Error bars depict the variation across different observation days and symbol–colour combinations represent different individuals.

Exploration rates increased steeply during the early dependency period, peaked at the age of around 2 years and subsequently decreased to reach near-zero values by the age of weaning (7–8.5 years, Figure 1).

### 3.2 Development of detailed aspects of dependent immatures’ exploration behaviour

**Figure 2.**
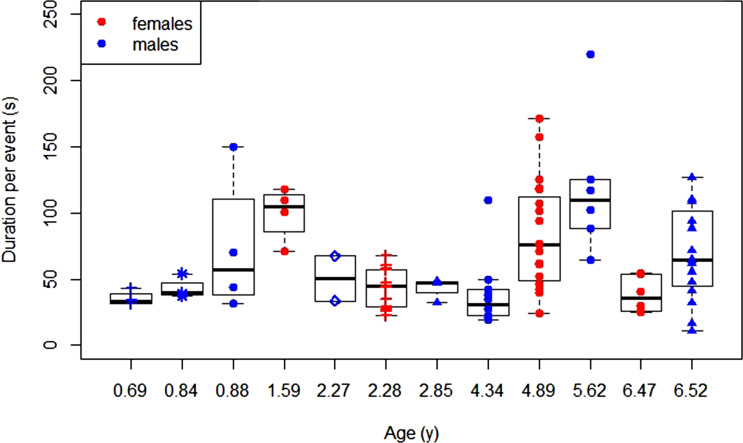
Development of exploration duration: daily average durations of exploration events over age for female and male dependent immatures for each data block with symbol–colour combinations representing different individuals.

Since exploratory tendency is highest during the dependency period, in the following we look at the detailed development of dependent immatures’ exploratory behaviour. In terms of exploration duration, we found that including the factor age did not significantly improve the model fit (likelihood ratio test: *χ*^2^ = 1.07, *p* = 0.301, Figure 2), suggesting that the average duration of the exploration events did not change over age.

**Figure 3.**
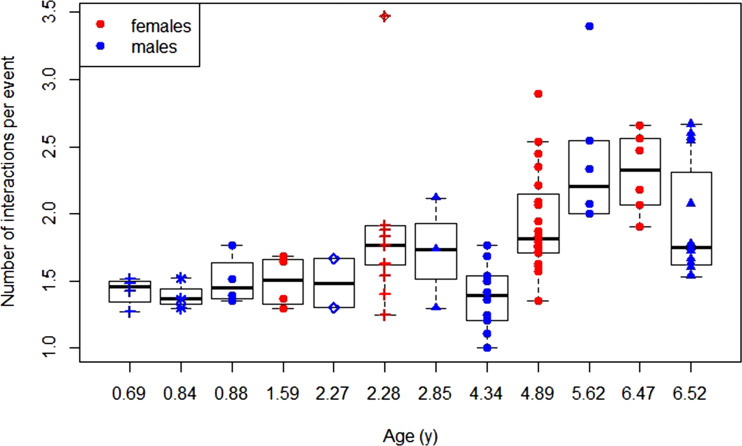
Development of exploratory manipulation action diversity: daily average number of exploratory actions performed per exploratory event over age for female and male dependent immatures for each data block with symbol–colour combinations representing different individuals.

When looking at the diversity of exploration behaviour as a function of age, we found that the model with age as a predictor variable was preferred over the null model LRT: *χ*^2^ = 11.73, *p =* 0.003). Furthermore, the model with age as a sigmoid factor was preferred over the model with age as linear factor (LRT: *χ*^2^ = 4.57, *p* < 0.001). The preferred model showed a significant positive effect of sigmoid age on exploration diversity (Table 1a, Figure 3).

**Table 1.** Effects of age on exploration diversity, number of body parts used and exploratory mouthing. Estimates, standard errors and *p*-values of the preferred full models. Significant effects of predictor variables are indicated in bold. For the model with the Gaussion family distribution, the *p*-values of the effects were obtained via the *cftest* function implemented in the *multcomp* package in R (Hothorn et al., 2016). *R*^2^ refers to conditional pseudo delta *R*^2^ values, obtained via the *MuMln* package (Bartoń, 2009; Nakagawa et al., 2017).

No.	Response	Factor	Factor type	Estimate	Standard error	*p*-Value	*R*^2^	Family distribution
(a)	Number of exploratory actions	Intercept	Intercept	−0.35	0.288	0.226	0.55	Poisson
		Sigmoid (Age)	Predictor	1.01	0.32	**0.002**		
		Log (Total exploration events)	Offset	—	—	—		
		Data block	Random	—	—	—		
		Individual	Random	—	—	—		
(b)	Number of body parts	Intercept	Intercept	−0.15	0.19	0.416	0.27	Poisson
		Age	Predictor	0.69	0.21	**<0.001**		
		Log (Total exploration events)	Offset	—	—	—		
		Data block	Random	—	—	—		
		Individual	Random	—	—	—		
(c)	Exploration events with mouthing	Intercept	Intercept	−0.17	0.05	0.001	0.13	Poisson
		Age	Predictor	−0.04	0.01	**0.005**		
		Log (Total exploration events)	Offset	—	—	—		
		Data block	Random	—	—	—		
		Individual	Random	—	—	—		

**Figure 4.**
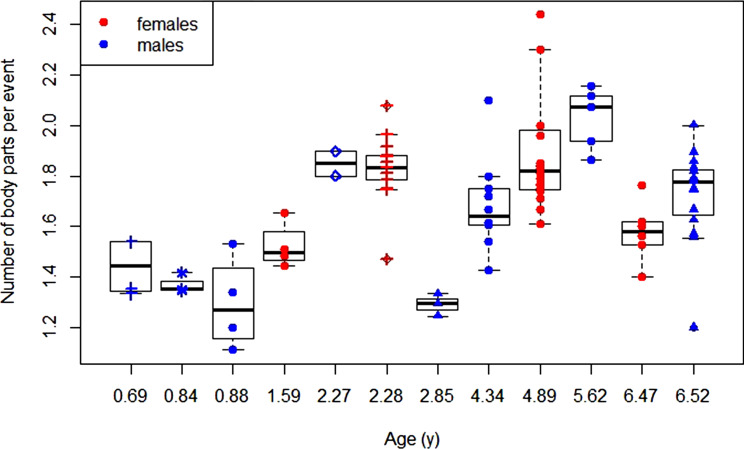
Development of exploratory body part diversity: daily average number of body parts used per exploratory event over age for female and male dependent immatures for each data block with symbol–colour combinations representing different individuals.

For the number of body parts used to manipulate the object we found that the model with age as a predictor variable was preferred over the null model (LRT: *χ*^2^ = 6.86, *p =* 0.009). Furthermore, the model with age as a sigmoid factor was preferred over the model with age as a linear factor (LRT: *χ*^2^ = 0.89, P < 0.001). The preferred model showed a significant positive effect of sigmoid age on the number of body parts involved in the exploration event (Table 1b, Figure 4).

**Figure 5.**
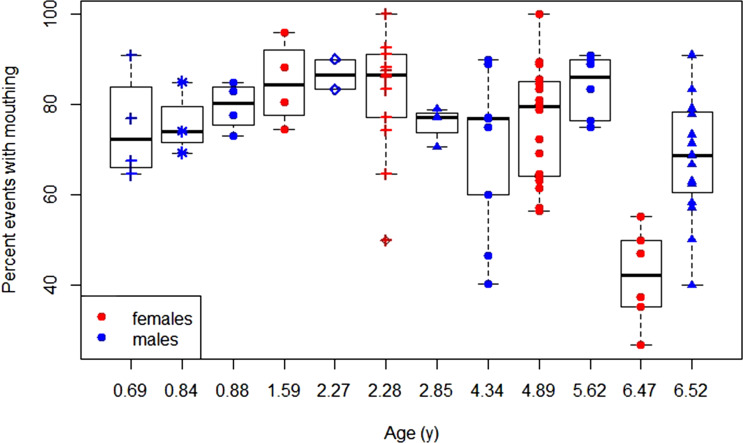
Development of exploratory mouthing: daily average shares of exploration events that involved the mouth (as a percentage of total exploration events) over age for female and male dependent immatures for each data block with symbol–colour combinations representing different individuals.

For the share of exploration events that included the mouth, we found that the model with age as a predictor variable was preferred over the null model (LRT: *χ*^2^ = 5.55, *p =* 0.019). The full model indicated that age had a significant negative effect on the share of exploration events that included the mouth (Table 1c, Figure 5). Model selection showed that including age as a sigmoid factor did not further increase the model fit.

## 4. Discussion

Our results showed several parallels between the development of exploratory object manipulation behaviour in orangutans and that in humans, suggesting that orangutans go through similar developmental trajectories of several aspects of exploration behaviour to humans. In general, developmental changes in exploration behaviour can be the result of advancing physical or cognitive development and it will be very difficult to tease these effects apart. Furthermore, even though similarities in developmental processes between closely related species suggest common ancestry as the underlying cause, comparisons of two species alone are limited in their conclusions. However, the similarities and differences in the development of orangutan and human exploration behaviour may help us to draw conclusions about the broad mechanisms underlying the development of the different aspects of exploratory behaviour.

In line with our prediction, our results showed that the overall age trajectory of immature orangutans’ exploratory objects manipulation rates is very similar to the human pattern, with a peak during the early dependency period (Belsky & Most, 1981; Bock, 2005; Power, 1999; Power et al., 1985). In wild chimpanzees, with increasing age, rates of object manipulation decrease while the manipulations become more goal-directed (Lamon et al., 2018). If exploratory object manipulations are an expression of learning processes, one would predict that humans show a higher exploration rate than orangutans and other great apes, owing to their advanced cognitive capacity. In a study in which human, chimpanzee and bonobo (*Pan pansicus*) infants were observed under a highly comparable free play setting in which they were provided with access to the same objects, human infants showed more frequent and more flexible object manipulations than chimpanzees and bonobos (Vauclair & Bard, 1983). Furthermore, chimpanzees were mouthing objects more frequently than bonobos and humans (Vauclair & Bard, 1983). Whereas experiments like this provide valuable insights into the propensity of the species to explore objects under identical conditions, data from everyday life in the wild are needed to assess the natural expression of exploration behaviour. Comparisons of levels of naturally occurring exploration would allow us to draw conclusions about the role of exploration in everyday learning. However, to our knowledge, there are only a handful of datasets available on the development of human infants’ rates of exploration behaviour during their everyday life and across contexts (but see: Bakeman et al., 1990; Belsky & Most, 1981; Bock, 2005; Pellegrini & Gustafson, 2005). Furthermore, while investigating species differences in infant exploration, studying human infants growing up in Western industrialized societies only might be misleading. Infants growing up in a larger variety of developmental contexts, including small-scale societies, would allow for the most meaningful comparison. Even though cross-cultural data are scarce, the available studies suggest similar patterns in the development of exploratory object manipulations across human societies (Bakeman et al., 1990; Bock, 2005; Crittenden, 2016; Gosso et al., 2005).

As predicted, in orangutans, just as in humans, with increasing age, exploration events become more variable in terms of the variety of manipulations and actions performed with the objects. This is not in line with the results of a previous study on object manipulation behaviour in orangutans (Bard, 1987), but that might be because the later study looked at a wider range of object manipulation behaviours than our study did. Furthermore, just as in humans, in orangutans, exploratory object mouthing (i.e., object manipulations with the mouth as opposed to with the fingers) decreased with increasing age. In humans, the increasing variety of manipulations and the increase in the number of object–object combinations used during exploratory object manipulations with age are an indicator of overall advancing cognitive development (Caruso, 1993; Jennings et al., 1979; McCall, 1974; McCall et al., 1972; Muentener et al., 2018; Power et al., 1985; Schuetze et al., 1999). Our results suggest that exploration may also be indicative of individuals’ advancing cognitive abilities in orangutans. These findings are consistent with the phylogenetic pattern of a correlated increase of manipulation complexity (defined via the number of object–object combinations and bimanual manipulations, and the number of body parts involved) with increasing levels of cognitive performance across species (Byrne et al., 2001; Hayashi & Matsuzawa, 2003; Heldstab et al., 2016; Power, 1999; Redshaw, 1978; Torigoe, 1985; Vauclair & Bard, 1983). The ontogenetic increase in object manipulation diversity over age is consistent with findings on wild chimpanzees whereas in wild bonobos there is no such age trend (Koops, Furuichi, Hashimoto, et al., 2015).

We predicted that, with increasing age, orangutans would become more persistent explorers and thus show longer exploration durations. However, our results showed that exploration durations did not increase over age. In humans, the results for the relationship between exploration duration and age are mixed: whereas some studies find a positive effect of age on exploration duration (Belsky et al., 1980), others don't (Power et al., 1985). However, across studies, human infants were found to become more efficient and competent explorers with increasing age (Belsky & Most, 1981; McCall, 1974; Muentener et al., 2018). An increase in efficiency and competence (e.g., owing to increasing motor skills and physical strength) may overrule any effect that an increase in exploration persistence may have on exploration duration because more efficient and competent individuals will reach their manipulation goals faster. The lack of increasing exploration duration with age may also be a result of time constraints that orangutan immatures in the wild experience. To test this hypothesis, one might look at the development of exploration persistence in zoo orangutans: zoo orangutans are expected to show fewer time constraints because of the reduced time that they spend foraging and the reduced need for vigilance (Kummer & Goodall, 1985).

The orangutan's arboreal lifestyle (Ashbury et al., 2015) may be one factor that affects object manipulations and may explain differences in object manipulation found between orangutans and humans as well as other non-human great apes. The orangutans at Suaq spend less than 0.1% of their time on the ground (Schuppli, personal communication), which probably limits their access to detached objects and may make bimanual manipulations more difficult. The arboreal lifestyle may also be an explanation for the high prevalence of mouthing in infant orangutans because they spend most of their time holding onto their mothers for the first two years of life and later onto tree branches. The decrease in exploratory mouthing may reflect an increase in the ability to use their hands while being in the trees because of overall increased motor skills (Van Noordwijk et al., 2009).

An obvious difference between humans and other great apes is that humans integrate a significantly wider variety of objects, including a larger variety of tools, into their everyday actions. In humans, objects are also frequently involved in mother–infant interactions, often in the form of object offerings by the mother to the infant (Bakeman et al., 1990), which is not the case in orangutans or other non-human great apes (Bard & Vauclair, 1984). Furthermore, there is an almost complete lack of maternal efforts to focus the infant's attention on objects to explore in wild orangutans, which is consistent with results for other non-human great apes in captivity (Bard & Vauclair, 1984). Non-human great ape mothers would be physically and most likely also cognitively capable of using attention-focusing behaviours (such as repositioning objects into the immature's reach; Belsky et al., 1980) as well as verbal attention-focusing behaviours in the form of simple calls (as all great ape species use calls in the mother–offspring context). However, within more than 10,000 hours of detailed mother–offspring observations at Suaq, spanning more than 13 years, we have only observed a handful of physical attention focusing events by mothers in the social learning context (during feeding and nest building). Similarly, we have only anecdotal reports of occasions where orangutan mothers seemed to use a ‘come-here’ call to attract their immatures to food sources. In short, whereas human mothers actively stimulate their infants’ exploratory behaviour (Belsky et al., 1980), orangutan mothers are much more passive. These findings are supported by similar findings for other non-human great apes in captivity (Bard & Vauclair, 1984).

In humans, aside from the active involvement of the mother in infants’ exploratory object manipulations, there are also more passive mechanisms at work: maternal attachment positively affects infants’ exploration behaviour (Main, 1983). Given that maternal attachment can be measured reliably in orangutans, the immediate and long-term effects of maternal attachment on exploratory tendency can be tested in a follow-up study, once sample sizes have increased.

Object manipulation is often described as an important developmental precursor of tool use (Bruner, 1972; Hayashi et al., 2006; Koops, Furuichi, & Hashimoto, 2015; Koops, Furuichi, Hashimoto, et al., 2015; McGrew, 1977). In several species, including chimpanzees, capuchin monkeys (*Cebus apella*) and macaques (*Macaca fascicularis*), detailed studies on the ontogeny of tool use behaviour showed that the underlying manipulative actions and in particular their appropriate sequential combinations develop gradually with age (De Resende et al., 2008; Inoue-Nakamura & Matsuzawa, 1997; Tan, 2017). Furthermore, there are species differences in the developmental trajectories of these manipulative tool use actions which are correlated with locomotor development and feeding ecology (De Resende et al., 2008). At Suaq, tool use is commonly performed by adult individuals in a large range of contexts (van Schaik et al., 2003; van Schaik & Knott, 2001). In line with this hypothesis, in a previous study, we showed that immature orangutans’ exploratory object manipulations were significantly higher at Suaq than at Tuanan, a different orangutan population without habitual tool use. This finding is further supported by differences in immatures’ object manipulations between wild chimpanzees and bonobos (Koops, Furuichi, & Hashimoto, 2015; Koops, Furuichi, Hashimoto, et al., 2015): in wild chimpanzees, who use tools in a variety of contexts, immatures show higher rates and more diverse types of object manipulation than bonobo immatures, which use few tools. Accordingly, obligate tool use in humans, in contrast to non-obligate tool use or the complete absence thereof great apes, may explain some of the differences in found in exploration frequency and complexity between human and great ape infants (Redshaw, 1978; Vauclair & Bard, 1983).

Also, in the context of object manipulations of immatures as preparation for tool use (or adult activity patterns in general), studies looked at sex differences in the predisposition of immatures to engage with objects. In humans, there seem to be no gender differences in infants’ exploration diversity (McCall, 1974; Muentener et al., 2018), but object play contexts differ in concordance with gender-specific adult activity patterns (e.g., playing with dolls in girls as preparation for mothering; Pellegrini & Bjorklund, 2004; Pellegrini & Gustafson, 2005; Pellegrini & Smith, 1998). In our study, we did not find any evidence for sex differences in any of the aspects of exploratory object manipulations analysed here. However, our data was unbalanced in terms of the age–sex distribution, which means that the negative result we find here need to be treated with caution. These findings are consistent with findings on wild immature bonobos but contrast with findings on wild immature chimpanzees, where males show higher rates of object manipulations than females (Koops, Furuichi, Hashimoto, et al., 2015; Lamon et al., 2018), whereas the opposite pattern was found for specific forms of object carrying (Kahlenberg & Wrangham, 2010). However, female chimpanzees develop their termite-fishing skills faster than males (Lonsdorf, 2005), despite faster motor development in males (Lonsdorf et al., 2014).

In summary, the similarity of the developmental patterns between wild orangutans’ and humans’ object exploration suggests that immature orangutans, like humans, learn about the world around them through the exploration of objects and that exploratory object manipulations may reflect cognitive development during immaturity. Differences in exploratory object manipulations between humans and non-human great apes and among the different great ape species are probably based on differences in lifestyle, ecology, the species reliance on tool use, and the amount and kind of social stimulation that individuals receive during development.

## Data Availability

The data associated with this research will be uploaded to a suitable data repository.

## Supplementary material

For supplementary material accompanying this paper visit https://doi.org/10.1017/ehs.2021.34. click here to view supplementary material
