# Supplementary material for: The ontogeny of exploratory object manipulation behaviour in wild orangutans
Source: Evol Hum Sci. 2021 Jul 2;3:e39. doi: 10.1017/ehs.2021.34 (PMC10427332; doi:10.1017/ehs.2021.34)
Supplement: Supplementary file 1 [file S2513843X21000347sup001.docx]

**Supplementary Material**

**The ontogeny of exploratory object manipulation behavior in wild orangutans**

Caroline Schuppli^1,2^_*_, AnaÏs Van Cauwenberghe^1,2^, Tatang Mirta Setia^3^, Daniel Haun^4^

*Corresponding author

1: Development and Evolution of Cognition Research Group, Max Planck Institute for Animal Behavior, Bücklestrasse 5a, 78467 Konstanz, Germany. Email: [caroline.schuppli@aim.uzh.ch](mailto:caroline.schuppli@aim.uzh.ch).

2: Department of Anthropology, University of Zürich, Winterthurerstrasse 190, 8057 Zürich, Switzerland. Email: [anaisvancauwenberghe@gmail.com](mailto:anaisvancauwenberghe@gmail.com).

3: Department of Biology, Graduate School and Faculty of Biology, Universitas Nasional, Jl. Sawo Manila, RT.14/RW.3, Ps. Minggu, DKI Jakarta, Indonesia. Email: [tatangmitra52@gmail.com](mailto:tatangmitra52@gmail.com).

4: Max Planck Institute for Evolutionary Anthropology, Deutscher Platz 6, 04103 Leipzig, Germany. Email: [haun@eva.mpg.de](mailto:haun@eva.mpg.de).

**Table S1. Explorative manipulations.** List of all explorative manipulations observed in this study, their definition, the youngest recorded age at first occurrence (in years), their total counted occurrence as well as their occurrence in percent (across all exploratory object manipulations in this study). Based on (Byrne et al., 2001; De Resende et al., 2008; Inoue-Nakamura & Matsuzawa, 1997; Torigoe, 1985)

| **Manipulation** | **Definition** | **Age at first occurrence (y)** | **Total occurence** | **Occurence percent** |
| --- | --- | --- | --- | --- |
| Bending | Shaping an object into a curve or angle | 0.69 | 24 | 0.57 |
| Biting | Cutting an object with teeth | 0.53 | 1004 | 23.64 |
| Breaking apart | Separate an object into two pieces | 0.88 | 99 | 2.33 |
| Carrying around | Transporting an object | 0.67 | 119 | 2.80 |
| Chewing | Repeatedly biting on an object that is inside the mouth | 0.53 | 375 | 8.83 |
| Digging | Moving hand or other body parts inside a substrate or object while breaking it up | 0.69 | 132 | 3.11 |
| Dropping | Make an object fall by letting go of it | 0.54 | 176 | 4.14 |
| Gnawing | Repeatedly biting on an object that is not inside the mouth | 0.69 | 126 | 2.97 |
| Hitting | Forcefully striking a body part against object | 0.69 | 45 | 1.06 |
| Kissing | Touching object with lips | 2.84 | 20 | 0.47 |
| Licking | Passing tongue over object | 0.92 | 38 | 0.89 |
| Licking hand | Passing tongue over hand after touching an object with the same hand | 1.58 | 17 | 0.40 |
| Nibbling | Gently repeatedly biting into an object while removing small pieces | 0.92 | 11 | 0.26 |
| Petting | Gently stroking over an object | 0.67 | 37 | 0.87 |
| Picking at | Repeatedly pulling at a small object | 0.69 | 58 | 1.37 |
| Poking | Jabbing finger into object | 0.68 | 251 | 5.91 |
| Pulling | Excreting force on object to cause movement towards actor | 0.54 | 198 | 4.66 |
| Pulling through mouth | Putting mouth around an object and then pulling the object through the mouth | 0.89 | 13 | 0.31 |
| Pushing | Excreting force on object to cause movement away from actor | 1.36 | 7 | 0.16 |
| Putting on head | Placing an object onto oned own head | 3.62 | 2 | 0.05 |
| Reaching into | Sticking body part (hand, arm, foot, head) into a substrate or object (e.g., tree hole or big piece of dead wood) | 1.58 | 159 | 3.74 |
| Ripping off | Tearing or pulling object off of a substrate or other object | 0.54 | 3 | 0.07 |
| Rubbing | Moving hands (or feet) over object while applying pressure | 4.31 | 23 | 0.54 |
| Scraping off | Removing small parts of the object by tearing them off | 2.29 | 284 | 6.69 |
| Scratching | Moving fingernails over object while applying pressure | 0.69 | 56 | 1.32 |
| Shaking | Rapidly moving a detached object up and down or from side to side | 0.88 | 15 | 0.35 |
| Slapping | Gently hitting an object with the palm of the hand | 2.29 | 4 | 0.09 |
| Smashing | Hitting an object against a surface | 2.85 | 119 | 2.80 |
| Smelling | Sniffing at an object | 0.69 | 7 | 0.16 |
| Sniffing Hand | Sniffing at hand after touching an object with the same hand | 0.92 | 3 | 0.07 |
| Spitting out | Forcefully ejecting object from mouth, making it fly away | 1.59 | 23 | 0.54 |
| Stripping off; Peeling | Removing covering from an object (e.g., bark from a twig) | 2.84 | 14 | 0.33 |
| Sucking | Drawing at an object with mouth | 0.53 | 40 | 0.94 |
| Swinging | Moving a suspended object back and forth or from side to side | 0.53 | 7 | 0.16 |
| Taking apart | Deconstructing composite objects (e.g., nests) by separating the parts | 2.84 | 17 | 0.40 |
| Taking in and out of mouth | Repeatedly taking object in and out of mouth | 0.67 | 199 | 4.69 |
| Tearing apart | Pulling objects into two pieces by applying force | 0.69 | 71 | 1.67 |
| Throwing | Propel object with force through the air | 2.84 | 21 | 0.49 |
| Tool use | Insert other object (mostly sticks) into explored object/ poking explored object with another object (mostly sticks) | 4.27 | 28 | 0.66 |
| Touching | Deliberately touching an object with hand (or foot), while looking at it intently but not performing any other actions with it | 0.69 | 153 | 3.60 |
| Turning around | Turning object around in hands and/ or feet. Often includes repeated handing of objects from hands to feet and back | 0.69 | 84 | 1.98 |
| Tuning in mouth | Turning an object around inside the mouth without chewing on it. Often includes looking at the object repeatedly on protruded lower lip | 1.59 | 17 | 0.40 |
| Twirling | Spinning and object quickly an lightly around, causing it to rotate | 4.86 | 3 | 0.07 |
| Waving around | Moving object in hand (or foot) through the air | 0.54 | 138 | 3.25 |
| Winding around | Wrapping a long object around another object or body part with twisting movements | 2.27 | 7 | 0.16 |

**Table S2. Body parts used during manipulations.** List of all body parts used during the explorative manipulations observed in this study and the age they were first used (in years).

| **Body part** | **Age at first occurrence (y)** |
| --- | --- |
| Finger | 0.87 |
| Fist | 0.88 |
| Foot | 3.69 |
| Hand | 0.54 |
| Head | 3.62 |
| Lip | 0.92 |
| Mouth | 0.53 |
| Nose | 0.69 |
| Shoulder | 4.31 |
| Teeth | 0.53 |
| Tongue | 0.92 |
| Upper Body (as a whole) | 6.5 |
| Wrist | 3.62 |

**Table S3. Focal individuals.** Sex, estimated date of birth, and the contributions to the two different data sets of all focal individuals of this study.

| **Name** | **Sex** | **Date of birth** | **Overall exploration rates (number of block data points)** | **Detailed Exploration data (number of daily data points)** |
| --- | --- | --- | --- | --- |
| Amor | Male | 01.01.2015 | 0 | 4 |
| Chindy | Female | 01.01.2003 | 1 | 0 |
| Cinnamon | Female | 01.04.2012 | 3 | 30 |
| Eden | Female | 01.11.2014 | 1 | 13 |
| Ellie | Female | 01.05.1999 | 1 | 0 |
| Frankie | Male | 01.08.2012 | 3 | 16 |
| Fredy | Male | 01.12.2004 | 2 | 0 |
| Lilly | Female | 01.03.2001 | 2 | 0 |
| Lois | Male | 01.08.2010 | 6 | 18 |
| Rendang | Male | 15.07.2013 | 1 | 4 |
| Shera | Female | 02.06.1998 | 1 | 0 |
| Simba | Male | 01.04.2013 | 1 | 4 |
| Tina | Female | 01.01.1998 | 1 | 0 |

**Table S4. Explorative manipulation combinations.** All observed combinations of all explorative manipulations during the study and their occurrence frequency (as total observed counts and in percent of all observed manipulations).

| **Manipulation combination** | **Occurence count** | **Occurence percent** |
| --- | --- | --- |
| Bending | 2 | 0.09 |
| Bending; Biting | 1 | 0.05 |
| Bending; Biting; CarryingAround | 1 | 0.05 |
| Bending; Biting; Pulling | 8 | 0.36 |
| Bending; Biting; TurningAround | 2 | 0.09 |
| Bending; Biting; TurningInMouth | 1 | 0.05 |
| Bending; CarryingAround; TakingIn&OutOfMouth; WindingAround | 1 | 0.05 |
| Bending; Chewing | 1 | 0.05 |
| Bending; Poking | 1 | 0.05 |
| Bending; Pulling | 2 | 0.09 |
| Bending; Pulling; Touching | 2 | 0.09 |
| Biting | 330 | 14.86 |
| Biting; BreakingApart | 13 | 0.59 |
| Biting; BreakingApart; CarryingAround | 1 | 0.05 |
| Biting; BreakingApart; Chewing | 1 | 0.05 |
| Biting; BreakingApart; Chewing; Gnawing; Twirling; WavingAround | 1 | 0.05 |
| Biting; BreakingApart; Chewing; WavingAround | 1 | 0.05 |
| Biting; BreakingApart; Dropping | 1 | 0.05 |
| Biting; BreakingApart; Dropping; Poking | 1 | 0.05 |
| Biting; BreakingApart; Dropping; Sucking | 2 | 0.09 |
| Biting; BreakingApart; Dropping; WavingAround | 3 | 0.14 |
| Biting; BreakingApart; Gnawing | 1 | 0.05 |
| Biting; BreakingApart; Gnawing; Licking; Poking; Scratching | 1 | 0.05 |
| Biting; BreakingApart; Poking | 1 | 0.05 |
| Biting; BreakingApart; Poking; WavingAround | 1 | 0.05 |
| Biting; BreakingApart; Scratching | 1 | 0.05 |
| Biting; BreakingApart; Scratching; Sucking | 1 | 0.05 |
| Biting; BreakingApart; TakingIn&OutOfMouth | 1 | 0.05 |
| Biting; BreakingApart; ToolUse | 1 | 0.05 |
| Biting; BreakingApart; TurningAround | 2 | 0.09 |
| Biting; BreakingApart; TurningAround; WavingAround | 1 | 0.05 |
| Biting; BreakingApart; WavingAround | 8 | 0.36 |
| Biting; CarryingAround | 9 | 0.41 |
| Biting; CarryingAround; Chewing | 1 | 0.05 |
| Biting; CarryingAround; Dropping; RippingOff; ToolUse; TurningAround | 1 | 0.05 |
| Biting; CarryingAround; Poking; Shaking | 2 | 0.09 |
| Biting; CarryingAround; Pulling | 1 | 0.05 |
| Biting; CarryingAround; Pulling; Pulling | 1 | 0.05 |
| Biting; CarryingAround; Pulling; RippingOff | 2 | 0.09 |
| Biting; CarryingAround; Pushing | 1 | 0.05 |
| Biting; CarryingAround; RippingOff | 3 | 0.14 |
| Biting; CarryingAround; TakingIn&OutOfMouth | 1 | 0.05 |
| Biting; CarryingAround; TurningInMouth | 1 | 0.05 |
| Biting; CarryingAround; WavingAround | 1 | 0.05 |
| Biting; Chewing | 43 | 1.94 |
| Biting; Chewing; Digging; Gnawing; Poking; Smelling | 1 | 0.05 |
| Biting; Chewing; Dropping | 8 | 0.36 |
| Biting; Chewing; Dropping; PickingAt | 3 | 0.14 |
| Biting; Chewing; Dropping; RippingOff | 12 | 0.54 |
| Biting; Chewing; Dropping; RippingOff; ScrapingOff | 10 | 0.45 |
| Biting; Chewing; Dropping; Scratching | 9 | 0.41 |
| Biting; Chewing; Gnawing; Poking | 1 | 0.05 |
| Biting; Chewing; Gnawing; RippingOff; Smelling | 1 | 0.05 |
| Biting; Chewing; Hitting | 2 | 0.09 |
| Biting; Chewing; PickingAt | 12 | 0.54 |
| Biting; Chewing; Pulling; RippingOff | 1 | 0.05 |
| Biting; Chewing; Pulling; TurningAround | 1 | 0.05 |
| Biting; Chewing; RippingOff | 6 | 0.27 |
| Biting; Chewing; RippingOff; WavingAround | 1 | 0.05 |
| Biting; Chewing; Scratching | 1 | 0.05 |
| Biting; Chewing; Sucking | 2 | 0.09 |
| Biting; Chewing; TakingIn&OutOfMouth | 2 | 0.09 |
| Biting; Chewing; TakingIn&OutOfMouth | 1 | 0.05 |
| Biting; Chewing; TearingApart | 1 | 0.05 |
| Biting; Chewing; TurningAround | 1 | 0.05 |
| Biting; Digging | 4 | 0.18 |
| Biting; Digging; Dropping; Gnawing; Pulling; Scratching; ReachingInto | 1 | 0.05 |
| Biting; Digging; Gnawing; LickingHand; Poking | 1 | 0.05 |
| Biting; Digging; Gnawing; Poking; Scratching | 1 | 0.05 |
| Biting; Digging; LickingHand; Poking; Scratching | 1 | 0.05 |
| Biting; Digging; LickingHand; Poking; Scratching; Smelling; Touching | 2 | 0.09 |
| Biting; Digging; Poking; Scratching | 2 | 0.09 |
| Biting; Digging; Pulling | 1 | 0.05 |
| Biting; Digging; Pushing; Scratching | 1 | 0.05 |
| Biting; Digging; Scratching | 3 | 0.14 |
| Biting; Digging; Scratching; ReachingInto | 1 | 0.05 |
| Biting; Digging; Scratching; Touching | 1 | 0.05 |
| Biting; Dropping | 7 | 0.32 |
| Biting; Dropping; Gnawing | 2 | 0.09 |
| Biting; Dropping; Gnawing; PickingAt | 8 | 0.36 |
| Biting; Dropping; Gnawing; RippingOff | 11 | 0.50 |
| Biting; Dropping; Gnawing; RippingOff; ScrapingOff | 8 | 0.36 |
| Biting; Dropping; Gnawing; WavingAround | 1 | 0.05 |
| Biting; Dropping; PickingAt | 3 | 0.14 |
| Biting; Dropping; Poking; ToolUse | 1 | 0.05 |
| Biting; Dropping; Pulling | 2 | 0.09 |
| Biting; Dropping; RippingOff | 4 | 0.18 |
| Biting; Dropping; RippingOff; ScrapingOff | 3 | 0.14 |
| Biting; Dropping; RippingOff; Smelling | 1 | 0.05 |
| Biting; Dropping; RippingOff; Swinging | 1 | 0.05 |
| Biting; Dropping; RippingOff; ToolUse | 2 | 0.09 |
| Biting; Dropping; RippingOff; WavingAround | 5 | 0.23 |
| Biting; Dropping; Scratching | 1 | 0.05 |
| Biting; Dropping; Scratching; TearingApart; WavingAround | 1 | 0.05 |
| Biting; Dropping; StrippingOff/Peeling | 1 | 0.05 |
| Biting; Dropping; TurningAround | 1 | 0.05 |
| Biting; Dropping; WavingAround | 2 | 0.09 |
| Biting; Gnawing | 10 | 0.45 |
| Biting; Gnawing; PickingAt | 7 | 0.32 |
| Biting; Gnawing; Poking | 1 | 0.05 |
| Biting; Gnawing; Poking; Scratching | 2 | 0.09 |
| Biting; Gnawing; Poking; Shaking; Touching | 1 | 0.05 |
| Biting; Gnawing; Poking; TearingApart | 1 | 0.05 |
| Biting; Gnawing; RippingOff | 1 | 0.05 |
| Biting; Gnawing; Scratching | 1 | 0.05 |
| Biting; Gnawing; StrippingOff/Peeling; TurningAround | 1 | 0.05 |
| Biting; Gnawing; TakingIn&OutOfMouth | 1 | 0.05 |
| Biting; Gnawing; TakingIn&OutOfMouth | 1 | 0.05 |
| Biting; Gnawing; TearingApart | 2 | 0.09 |
| Biting; Gnawing; TurningAround | 3 | 0.14 |
| Biting; Gnawing; TurningInMouth | 1 | 0.05 |
| Biting; Gnawing; WavingAround; WindingAround | 1 | 0.05 |
| Biting; Hitting | 9 | 0.41 |
| Biting; Hitting; Pulling | 2 | 0.09 |
| Biting; Hitting; Touching | 1 | 0.05 |
| Biting; Kissing | 3 | 0.14 |
| Biting; Kissing; TearingApart | 1 | 0.05 |
| Biting; Licking | 1 | 0.05 |
| Biting; Licking; Poking | 1 | 0.05 |
| Biting; Licking; Smelling | 3 | 0.14 |
| Biting; LickingHand; Poking; Scratching; Smelling; Touching | 1 | 0.05 |
| Biting; Nibbling | 2 | 0.09 |
| Biting; Petting; Poking | 1 | 0.05 |
| Biting; PickingAt | 1 | 0.05 |
| Biting; Poking | 5 | 0.23 |
| Biting; Poking; RippingOff | 1 | 0.05 |
| Biting; Poking; RippingOff; Smelling | 1 | 0.05 |
| Biting; Poking; Scratching | 23 | 1.04 |
| Biting; Poking; Scratching; Smashing | 2 | 0.09 |
| Biting; Poking; Scratching; Sucking | 1 | 0.05 |
| Biting; Poking; Scratching; Sucking; TurningAround | 1 | 0.05 |
| Biting; Poking; Scratching; TearingApart | 1 | 0.05 |
| Biting; Poking; Shaking | 39 | 1.76 |
| Biting; Poking; Smelling | 3 | 0.14 |
| Biting; Poking; Sucking | 2 | 0.09 |
| Biting; Poking; Sucking; Touching | 1 | 0.05 |
| Biting; Poking; Sucking; WavingAround | 1 | 0.05 |
| Biting; Poking; TearingApart; WavingAround | 1 | 0.05 |
| Biting; Poking; ToolUse; TurningAround | 1 | 0.05 |
| Biting; Poking; TurningAround | 1 | 0.05 |
| Biting; Pulling | 50 | 2.25 |
| Biting; Pulling; Pulling | 3 | 0.14 |
| Biting; Pulling; Pulling; TurningAround; TurningAround | 1 | 0.05 |
| Biting; Pulling; Pulling; Twirling; Twirling | 1 | 0.05 |
| Biting; Pulling; Pushing; Slapping | 1 | 0.05 |
| Biting; Pulling; RippingOff | 2 | 0.09 |
| Biting; Pulling; Scratching | 1 | 0.05 |
| Biting; Pulling; Shaking | 2 | 0.09 |
| Biting; Pulling; Smelling | 2 | 0.09 |
| Biting; Pulling; StrippingOff/Peeling; TearingApart; TurningAround | 1 | 0.05 |
| Biting; Pulling; Swinging | 1 | 0.05 |
| Biting; Pulling; TearingApart | 2 | 0.09 |
| Biting; Pulling; Touching | 3 | 0.14 |
| Biting; Pulling; TurningAround | 5 | 0.23 |
| Biting; Pulling; TurningAround; WavingAround; WindingAround | 1 | 0.05 |
| Biting; Pulling; WavingAround | 4 | 0.18 |
| Biting; PullingThroughMouth | 1 | 0.05 |
| Biting; PullingThroughMouth ; RippingOff | 1 | 0.05 |
| Biting; RippingOff | 9 | 0.41 |
| Biting; RippingOff; ScrapingOff | 1 | 0.05 |
| Biting; RippingOff; TearingApart; WavingAround | 1 | 0.05 |
| Biting; RippingOff; Throwing; WavingAround | 1 | 0.05 |
| Biting; RippingOff; ToolUse | 1 | 0.05 |
| Biting; RippingOff; TurningAround | 1 | 0.05 |
| Biting; Scratching | 13 | 0.59 |
| Biting; Scratching; Smelling | 2 | 0.09 |
| Biting; Scratching; Smelling; Touching | 1 | 0.05 |
| Biting; Scratching; TakingIn&OutOfMouth; TurningAround | 1 | 0.05 |
| Biting; Scratching; Touching | 3 | 0.14 |
| Biting; Shaking | 2 | 0.09 |
| Biting; Slapping | 3 | 0.14 |
| Biting; Smelling | 7 | 0.32 |
| Biting; Smelling; TearingApart | 1 | 0.05 |
| Biting; SmellingHand; SmellingHand; Touching | 1 | 0.05 |
| Biting; SpittingOut | 1 | 0.05 |
| Biting; ReachingInto | 1 | 0.05 |
| Biting; StrippingOff/Peeling | 1 | 0.05 |
| Biting; Sucking | 8 | 0.36 |
| Biting; TakingApart | 2 | 0.09 |
| Biting; TakingIn&OutOfMouth | 16 | 0.72 |
| Biting; TakingIn&OutOfMouth | 1 | 0.05 |
| Biting; TakingIn&OutOfMouth ; TearingApart | 1 | 0.05 |
| Biting; TakingIn&OutOfMouth; TearingApart | 3 | 0.14 |
| Biting; TearingApart | 14 | 0.63 |
| Biting; TearingApart; WavingAround | 2 | 0.09 |
| Biting; ToolUse | 2 | 0.09 |
| Biting; Touching | 2 | 0.09 |
| Biting; TurningAround | 11 | 0.50 |
| Biting; TurningAround; TurningInMouth | 1 | 0.05 |
| Biting; TurningAround; WavingAround | 3 | 0.14 |
| Biting; TurningInMouth | 3 | 0.14 |
| Biting; WavingAround | 31 | 1.40 |
| BreakingApart | 3 | 0.14 |
| BreakingApart; Biting; Gnawing; TearingApart; Throwing | 2 | 0.09 |
| BreakingApart; CarryingAround; Dropping; StrippingOff/Peeling; ToolUse | 1 | 0.05 |
| BreakingApart; CarryingAround; Gnawing; PuttingOnHead; WavingAround | 1 | 0.05 |
| BreakingApart; CarryingAround; ToolUse | 2 | 0.09 |
| BreakingApart; Chewing | 2 | 0.09 |
| BreakingApart; Chewing; Poking | 1 | 0.05 |
| BreakingApart; Chewing; Poking; Shaking; StrippingOff/Peeling; ToolUse | 1 | 0.05 |
| BreakingApart; Chewing; StrippingOff/Peeling; ToolUse | 1 | 0.05 |
| BreakingApart; Chewing; Sucking | 1 | 0.05 |
| BreakingApart; Digging | 1 | 0.05 |
| BreakingApart; Digging; Dropping | 1 | 0.05 |
| BreakingApart; Digging; Peeling; Poking; Scratching; ReachingInto | 1 | 0.05 |
| BreakingApart; Dropping | 8 | 0.36 |
| BreakingApart; Dropping; Pulling; ToolUse | 1 | 0.05 |
| BreakingApart; Dropping; Scratching | 1 | 0.05 |
| BreakingApart; Dropping; StrippingOff/Peeling | 2 | 0.09 |
| BreakingApart; Dropping; Sucking | 2 | 0.09 |
| BreakingApart; Dropping; TakingIn&OutOfMouth; WavingAround | 1 | 0.05 |
| BreakingApart; Dropping; WavingAround | 2 | 0.09 |
| BreakingApart; Gnawing; Poking | 1 | 0.05 |
| BreakingApart; PuttingOnHead; WavingAround | 1 | 0.05 |
| BreakingApart; Scratching | 1 | 0.05 |
| BreakingApart; Sucking | 2 | 0.09 |
| BreakingApart; Throwing | 3 | 0.14 |
| BreakingApart; ToolUse | 4 | 0.18 |
| BreakingApart; TurningAround | 1 | 0.05 |
| BreakingApart; TurningAround; WavingAround | 1 | 0.05 |
| BreakingApart; WavingAround | 3 | 0.14 |
| CarryingAround | 54 | 2.43 |
| CarryingAround; Chewing | 13 | 0.59 |
| CarryingAround; Chewing; TakingIn&OutOfMouth | 1 | 0.05 |
| CarryingAround; Chewing; TakingIn&OutOfMouth | 1 | 0.05 |
| CarryingAround; Digging | 1 | 0.05 |
| CarryingAround; Dropping; TurningAround | 1 | 0.05 |
| CarryingAround; Nibbling | 3 | 0.14 |
| CarryingAround; RippingOff; StrippingOff/Peeling; TearingApart; ToolUse | 1 | 0.05 |
| CarryingAround; RippingOff; Touching | 1 | 0.05 |
| CarryingAround; TakingIn&OutOfMouth | 12 | 0.54 |
| CarryingAround; WavingAround | 3 | 0.14 |
| Chewing | 122 | 5.50 |
| Chewing; Digging; Poking; Scratching; TearingApart; WavingAround | 1 | 0.05 |
| Chewing; Dropping | 2 | 0.09 |
| Chewing; Dropping; RippingOff | 1 | 0.05 |
| Chewing; Dropping; RippingOff; Scratching | 3 | 0.14 |
| Chewing; Dropping; RippingOff; TearingApart | 3 | 0.14 |
| Chewing; Dropping; TakingIn&OutOfMouth | 1 | 0.05 |
| Chewing; Hitting | 1 | 0.05 |
| Chewing; Licking | 2 | 0.09 |
| Chewing; Nibbling; RippingOff | 1 | 0.05 |
| Chewing; PickingAt | 12 | 0.54 |
| Chewing; PickingAt; Sucking | 2 | 0.09 |
| Chewing; PickingAt; WavingAround | 1 | 0.05 |
| Chewing; Poking; RippingOff | 1 | 0.05 |
| Chewing; Poking; Scratching | 1 | 0.05 |
| Chewing; Pulling | 3 | 0.14 |
| Chewing; Pulling; PullingThroughMouth ; WavingAround | 1 | 0.05 |
| Chewing; Pulling; RippingOff | 4 | 0.18 |
| Chewing; PullingThroughMouth | 2 | 0.09 |
| Chewing; PullingThroughMouth | 1 | 0.05 |
| Chewing; RippingOff | 13 | 0.59 |
| Chewing; RippingOff; Smelling | 2 | 0.09 |
| Chewing; RippingOff; TakingIn&OutOfMouth | 7 | 0.32 |
| Chewing; RippingOff; TakingIn&OutOfMouth | 8 | 0.36 |
| Chewing; RippingOff; TakingIn&OutOfMouth; WavingAround | 1 | 0.05 |
| Chewing; SpittingOut | 2 | 0.09 |
| Chewing; Sucking | 2 | 0.09 |
| Chewing; TakingIn&OutOfMouth | 42 | 1.89 |
| Chewing; TakingIn&OutOfMouth; TurningInMouth | 1 | 0.05 |
| Chewing; Touching; TurningAround | 1 | 0.05 |
| Chewing; TurningAround | 2 | 0.09 |
| Chewing; TurningInMouth | 2 | 0.09 |
| Chewing; WavingAround | 1 | 0.05 |
| Digging | 58 | 2.61 |
| Digging; Gnawing; Scratching | 1 | 0.05 |
| Digging; Hitting | 1 | 0.05 |
| Digging; Hitting; TearingApart | 1 | 0.05 |
| Digging; Licking | 4 | 0.18 |
| Digging; Licking; Poking; Scratching; Smelling | 1 | 0.05 |
| Digging; LickingHand; Poking; Scratching | 1 | 0.05 |
| Digging; LickingHand; SmellingHand; Touching | 1 | 0.05 |
| Digging; Poking | 5 | 0.23 |
| Digging; Poking; Scratching | 8 | 0.36 |
| Digging; Poking; Scratching; Smelling | 1 | 0.05 |
| Digging; Pulling | 7 | 0.32 |
| Digging; Pushing | 1 | 0.05 |
| Digging; Scratching | 12 | 0.54 |
| Digging; Scratching; Touching | 1 | 0.05 |
| Digging; Smelling | 1 | 0.05 |
| Digging; StrippingOff/Peeling | 1 | 0.05 |
| Digging; TakingApart | 4 | 0.18 |
| Digging; Throwing | 2 | 0.09 |
| Digging; Touching | 2 | 0.09 |
| Dropping | 2 | 0.09 |
| Dropping; RippingOff | 7 | 0.32 |
| Dropping; RippingOff; Scratching; Smelling | 1 | 0.05 |
| Dropping; RippingOff; TakingIn&OutOfMouth; TurningAround; WavingAround | 3 | 0.14 |
| Dropping; RippingOff; Touching | 1 | 0.05 |
| Dropping; Scratching | 3 | 0.14 |
| Dropping; Scratching; Smelling; ReachingInto | 1 | 0.05 |
| Dropping; Scratching; ReachingInto | 2 | 0.09 |
| Dropping; Scratching; TakingIn&OutOfMouth | 6 | 0.27 |
| Dropping; Scratching; TearingApart | 1 | 0.05 |
| Dropping; Smelling | 1 | 0.05 |
| Dropping; TakingApart | 2 | 0.09 |
| Dropping; TakingIn&OutOfMouth; TurningInMouth | 1 | 0.05 |
| Dropping; TearingApart | 2 | 0.09 |
| Gnawing | 7 | 0.32 |
| Gnawing; Biting | 3 | 0.14 |
| Gnawing; CarryingAround; Nibbling | 1 | 0.05 |
| Gnawing; Hitting; Pulling | 2 | 0.09 |
| Gnawing; Licking | 1 | 0.05 |
| Gnawing; Licking; PickingAt; Poking; Scratching | 1 | 0.05 |
| Gnawing; Licking; Poking; Scratching | 1 | 0.05 |
| Gnawing; Licking; Poking; Sucking | 1 | 0.05 |
| Gnawing; LickingHand; Poking; Scratching | 1 | 0.05 |
| Gnawing; Nibbling | 1 | 0.05 |
| Gnawing; Poking | 6 | 0.27 |
| Gnawing; Poking; ScrapingOff | 1 | 0.05 |
| Gnawing; Poking; Scratching | 7 | 0.32 |
| Gnawing; Pulling | 3 | 0.14 |
| Gnawing; Pulling; Scratching | 1 | 0.05 |
| Gnawing; Scratching | 7 | 0.32 |
| Gnawing; Scratching; Smelling | 1 | 0.05 |
| Gnawing; Scratching; Touching | 1 | 0.05 |
| Gnawing; Smelling | 2 | 0.09 |
| Gnawing; TakingIn&OutOfMouth | 6 | 0.27 |
| Gnawing; Touching | 2 | 0.09 |
| Gnawing; WavingAround | 1 | 0.05 |
| Hitting | 15 | 0.68 |
| Hitting; Licking | 1 | 0.05 |
| Hitting; Petting; Pulling | 1 | 0.05 |
| Hitting; Poking | 2 | 0.09 |
| Hitting; Pulling | 4 | 0.18 |
| Hitting; Pulling; Smelling | 1 | 0.05 |
| Hitting; Pulling; TurningAround | 1 | 0.05 |
| Hitting; RippingOff | 1 | 0.05 |
| Hitting; Touching | 1 | 0.05 |
| Kissing | 9 | 0.41 |
| Kissing; Licking; Scratching; Touching | 1 | 0.05 |
| Kissing; Poking; Touching | 1 | 0.05 |
| Kissing; Scratching | 2 | 0.09 |
| Kissing; Smelling; TearingApart; Touching | 1 | 0.05 |
| Kissing; Smelling; Touching | 1 | 0.05 |
| Kissing; Touching | 1 | 0.05 |
| Licking | 3 | 0.14 |
| Licking; Poking | 1 | 0.05 |
| Licking; Poking; Scratching | 2 | 0.09 |
| Licking; Poking; Scratching; Smelling | 1 | 0.05 |
| Licking; Poking; Smelling | 1 | 0.05 |
| Licking; Poking; Touching | 1 | 0.05 |
| Licking; Pulling; Scratching | 1 | 0.05 |
| Licking; RippingOff; Rubbing; Smelling | 1 | 0.05 |
| Licking; RippingOff; Smelling | 1 | 0.05 |
| Licking; Scratching; Touching | 2 | 0.09 |
| Licking; Smelling | 1 | 0.05 |
| Licking; Smelling; Touching | 3 | 0.14 |
| Licking; Sucking | 1 | 0.05 |
| LickingHand | 2 | 0.09 |
| LickingHand; Poking; Scratching | 1 | 0.05 |
| LickingHand; Smelling; Touching | 1 | 0.05 |
| LickingHand; SmellingHand; Touching | 1 | 0.05 |
| LickingHand; Touching | 4 | 0.18 |
| Nibbling | 3 | 0.14 |
| Petting | 12 | 0.54 |
| Petting; Poking | 10 | 0.45 |
| Petting; Poking; Smelling | 2 | 0.09 |
| Petting; Poking; Touching | 1 | 0.05 |
| Petting; Scratching; Touching | 1 | 0.05 |
| Petting; Smelling | 3 | 0.14 |
| Petting; Touching | 6 | 0.27 |
| PickingAt | 6 | 0.27 |
| PickingAt; TakingIn&OutOfMouth | 1 | 0.05 |
| PickingAt; ToolUse | 1 | 0.05 |
| Poking | 30 | 1.35 |
| Poking; Pulling | 1 | 0.05 |
| Poking; RippingOff; Scratching | 1 | 0.05 |
| Poking; Scratching | 44 | 1.98 |
| Poking; Scratching; Smashing | 2 | 0.09 |
| Poking; Scratching; Smelling | 5 | 0.23 |
| Poking; Scratching; TakingIn&OutOfMouth; Touching | 1 | 0.05 |
| Poking; Scratching; TearingApart | 2 | 0.09 |
| Poking; Scratching; Throwing | 1 | 0.05 |
| Poking; Scratching; Touching | 2 | 0.09 |
| Poking; Slapping | 1 | 0.05 |
| Poking; Smelling | 6 | 0.27 |
| Poking; Smelling; Touching | 1 | 0.05 |
| Poking; SmellingHand; ReachingInto | 1 | 0.05 |
| Poking; ReachingInto | 6 | 0.27 |
| Poking; Sucking | 1 | 0.05 |
| Poking; TearingApart | 1 | 0.05 |
| Poking; ToolUse | 2 | 0.09 |
| Poking; ToolUse; Touching | 1 | 0.05 |
| Poking; Touching | 6 | 0.27 |
| Poking; WavingAround | 1 | 0.05 |
| Pulling | 19 | 0.86 |
| Pulling; Pushing | 1 | 0.05 |
| Pulling; Pushing; Scratching; ReachingInto; Swinging; Touching; WavingAround | 1 | 0.05 |
| Pulling; Scratching | 2 | 0.09 |
| Pulling; Shaking | 1 | 0.05 |
| Pulling; Slapping | 1 | 0.05 |
| Pulling; Smelling | 1 | 0.05 |
| Pulling; Sucking | 1 | 0.05 |
| Pulling; TakingIn&OutOfMouth | 1 | 0.05 |
| Pulling; TearingApart | 1 | 0.05 |
| Pulling; Touching | 14 | 0.63 |
| Pulling; TurningAround | 8 | 0.36 |
| Pulling; TurningAround; WavingAround | 2 | 0.09 |
| Pulling; WavingAround | 1 | 0.05 |
| PullingThroughMouth | 5 | 0.23 |
| PullingThroughMouth; TearingApart; WindingAround | 1 | 0.05 |
| Pushing; Touching | 1 | 0.05 |
| RippingOff | 4 | 0.18 |
| RippingOff; Rubbing; Scratching | 1 | 0.05 |
| RippingOff; Smelling | 1 | 0.05 |
| RippingOff; Smelling; TearingApart | 1 | 0.05 |
| RippingOff; TakingIn&OutOfMouth; TurningAround | 1 | 0.05 |
| RippingOff; TakingIn&OutOfMouth; WavingAround | 2 | 0.09 |
| RippingOff; ToolUse | 3 | 0.14 |
| Rubbing; Scratching | 1 | 0.05 |
| Scratching | 59 | 2.66 |
| Scratching; Shaking | 1 | 0.05 |
| Scratching; Slapping | 1 | 0.05 |
| Scratching; Smelling | 8 | 0.36 |
| Scratching; ReachingInto | 3 | 0.14 |
| Scratching; StrippingOff/Peeling | 1 | 0.05 |
| Scratching; TakingApart | 1 | 0.05 |
| Scratching; TearingApart; Touching | 1 | 0.05 |
| Scratching; Throwing | 1 | 0.05 |
| Scratching; Touching | 9 | 0.41 |
| Shaking | 1 | 0.05 |
| Shaking; Biting; BreakingApart | 1 | 0.05 |
| Shaking; Biting; Hitting; Pulling | 1 | 0.05 |
| Shaking; Chewing; Pulling | 1 | 0.05 |
| Slapping | 7 | 0.32 |
| Smelling | 11 | 0.50 |
| Smelling; Poking | 1 | 0.05 |
| Smelling; Pulling | 1 | 0.05 |
| Smelling; SmellingHand | 1 | 0.05 |
| Smelling; ReachingInto | 1 | 0.05 |
| Smelling; TakingIn&OutOfMouth | 5 | 0.23 |
| Smelling; TearingApart | 2 | 0.09 |
| Smelling; Touching | 16 | 0.72 |
| Smelling; TurningAround | 3 | 0.14 |
| Smelling; TurningAround; WavingAround | 1 | 0.05 |
| SmellingHand; Touching | 2 | 0.09 |
| ReachingInto | 4 | 0.18 |
| StrippingOff/Peeling | 1 | 0.05 |
| Sucking | 6 | 0.27 |
| Sucking; TakingIn&OutOfMouth | 1 | 0.05 |
| Swinging | 4 | 0.18 |
| TakingApart | 3 | 0.14 |
| TakingApart; TearingApart | 1 | 0.05 |
| TakingApart; Throwing | 3 | 0.14 |
| TakingApart; Throwing; WavingAround | 1 | 0.05 |
| TakingIn&OutOfMouth | 62 | 2.79 |
| TakingIn&OutOfMouth; TearingApart | 2 | 0.09 |
| TakingIn&OutOfMouth; TurningAround | 2 | 0.09 |
| TakingIn&OutOfMouth; TurningInMouth | 2 | 0.09 |
| TakingIn&OutOfMouth; WavingAround | 1 | 0.05 |
| TearingApart | 15 | 0.68 |
| Throwing | 6 | 0.27 |
| ToolUse | 2 | 0.09 |
| Touching | 43 | 1.94 |
| TurningAround | 10 | 0.45 |
| TurningAround; WavingAround | 2 | 0.09 |
| TurningInMouth | 3 | 0.14 |
| WavingAround | 4 | 0.18 |

**References**

Byrne, R. W., Corp, N., & Byrne, J. M. (2001). Manual dexterity in the gorilla: bimanual and digit role differentiation in a natural task. *Anim. Cog.*, *4*(3-4), 347-361.

De Resende, B. D., Ottoni, E. B., & Fragaszy, D. M. (2008). Ontogeny of manipulative behavior and nut‐cracking in young tufted capuchin monkeys (*Cebus apella*): a Perception–action perspective. *Dev. Sci.*, *11*(6), 828-840.

Inoue-Nakamura, N., & Matsuzawa, T. (1997). Development of stone tool use by wild chimpanzees (*Pan troglodytes*). *J. Comp. Psychol.*, *111*(2), 159.

Torigoe, T. (1985). Comparison of object manipulation among 74 species of non-human primates. *Primates*, *26*(2), 182-194.
